# Testing the effect of ecolabels on the environmental impact of food purchases in worksite cafeterias: a randomised controlled trial

**DOI:** 10.1186/s12889-024-21272-4

**Published:** 2025-01-11

**Authors:** Madison Luick, Cristina Stewart, Michael Clark, Paul Bateman, Elizabeth Biggs, Brian Cook, Melissa Little, Gina M. Wren, Susan A. Jebb, Rachel Pechey

**Affiliations:** 1 Nuffield Department of Primary Care Health Sciences, University of Oxford, Radcliffe Observatory Quarter, Woodstock Road, Oxford, OX2 6GG UK; 2 Nuffield Department of Population Health, University of Oxford, Oxford, UK; 3 Smith School of Enterprise and the Environment, University of Oxford, Oxford, UK; 4 Oxford Martin School, University of Oxford, Oxford, UK; 5 Department of Biology, University of Oxford, University of Oxford, Oxford, UK; 6 MRC/CSO Social and Public Health Sciences Unit, School of Health and Wellbeing, University of Glasgow, Glasgow, UK

**Keywords:** Ecolabels, Consumer behaviour, Purchasing, Food, RCT

## Abstract

**Background:**

Reducing the environmental impact of foods consumed is important for meeting climate goals. We aimed to conduct a randomised controlled trial to test whether ecolabels reduce the environmental impact of food selected in worksite cafeterias, alone or in combination with increased availability of more sustainable meal options.

**Methods:**

Worksite cafeterias (*n* = 96) were randomised to one of three study groups, with 54 included for final analysis. One group was intended to increase the availability of meat-free options, but no change was implemented. Therefore, this group was treated as part of the control, creating two groups: (1) control (no ecolabels) (*n* = 35), and (2) ecolabels (*n* = 19). Regression analysis assessed the primary outcome of total environmental impact of hot meals sold over a 6-week period. Secondary outcome analyses explored the individual environmental indicators that composed the total environmental impact score (i.e., greenhouse gas emissions, biodiversity loss, eutrophication, and water scarcity). The mean weekly environmental impact scores of hot meal options over the full 12-week trial period were assessed using hierarchical mixed effects models.

**Results:**

There was no significant effect of the intervention on the environmental impact scores of meals sold (mean difference between control and intervention sites: -1.4%, 95%CI: -33.6%, + 30.8%). There was no evidence of an effect in mean weekly environmental impact score (-5.4%, 95%CI: -12.6%, + 2.5%), nor in any of the four individual environmental indicators (greenhouse gas emissions: -3.6%, 95%CI: -30.7%, 34.3%; biodiversity loss: 2.0%, 95%CI: -25.8%, 40.2%; eutrophication: -2.4%, 95%CI: -29.3%, 34.7%; water scarcity: -0.4%, 95%CI: -28.7%, 39.1%).

**Conclusions:**

Ecolabels may not be an effective tool to shift consumer behaviour in worksite cafeterias towards meals with lower environmental impact.

**Trial registration:**

The study was pre-registered prospectively on ISRCTN (https://www.isrctn.com/ISRCTN10268258; 06/01/2022).

**Supplementary Information:**

The online version contains supplementary material available at 10.1186/s12889-024-21272-4.

## Background

A rapid reduction in the environmental impacts associated with food consumption is needed to meet global environmental targets [1]. Changing dietary behaviour – and reducing meat consumption in particular – will be key to this transition. However, changing dietary behaviours is difficult, even for those motivated to do so. For instance, although more than half of UK consumers have stated they want to change their diets to be more environmentally sustainable [2], reported consumption of meat in the UK has not changed in line with suggested targets – with reports suggesting consumption is decreasing only slowly (-17% in a recent decade) [3] or even increasing [4]. Some of the difficulty in changing dietary habits could be explained by the fact that consumers often do not realise the environmental impact of the foods they consume [5, 6]. This change may be made easier if food environments actively supported people to make more environmentally sustainable food choices.

Environmental sustainability labels (ecolabels) are increasingly used by businesses to inform consumers about the environmental impact of their food choices, and make the opportunity to select lower impact foods more salient. They have been discussed as viable policy options, with plans considered in some places, such as France, to implement ecolabels on food in supermarkets, with the aim of helping inform consumers to make more sustainable food choices [7]. In the UK, discussions as part of the Food Data Transparency Partnership have included the consideration of ecolabels on foods, and specifically, the development of a uniform methodology [8].

Systematic reviews have concluded that labels signifying a more sustainable choice could positively influence the selection, purchase and consumption of food and drink products [9, 10]. However, one review was dominated by ‘organic’ labels which may have prompted selection based on values beyond sustainable eating patterns, such as concerns about pesticide residues, and were beyond the scope of this research [10]. The second review noted the need for more field trials in this area since most studies have been conducted under experimental, sometimes hypothetical, conditions [9]. This is especially important to inform future policy options. One experimental study in university cafeterias found ecolabels decreased the probability of selecting high-carbon meals [11], but a field study in worksite cafeterias found no evidence of an effect of ecolabels on the environmental impact of purchases [12]. However, in this worksite cafeteria field study there were limited meal options rated as lower impact, potentially masking the potential for impact from ecolabels on consumer behaviour.

Increasing the availability of healthier or more sustainable options has been previously identified as a potentially important intervention. A Cochrane review found that increasing the availability of specific food options can increase their selection, albeit with low overall certainty [13]. Observational data has shown a positive association between increased meat-free meal availability and higher meat-free purchasing [14, 15]. Moreover, a field study in a university cafeteria demonstrated that doubling the proportion of vegetarian meals led to an approximately 40% rise in vegetarian meal sales [15]. Typically, meat-free items are lower impact [16, 17], and these findings suggest that increasing the availability of meat-free meals may be a promising strategy to reduce the overall environmental impact of meal choices.

This randomised controlled trial (RCT) aimed to further test the effectiveness of these interventions to encourage more sustainable food purchasing. The objective was to investigate the impacts of (a) ecolabels, (b) increased availability of meat-free options (herein: meat-free availability), and (c) the combined impact of implementing both interventions simultaneously, on the environmental impact of hot meal items purchased in worksite cafeterias, hypothesising that ecolabels may be more effective when accompanied by increased availability of meat-free meals. Unfortunately, availability was not increased as planned. As a result, the only objective studied here is the impact of ecolabels on the environmental impact of hot meal items purchased.

## Methods

### Study design and setting

Between January and April 2022, we conducted a 12-week RCT in worksite cafeterias run by a single operator, with sites using different menus and pricing structures based on the workplace (e.g. distribution or manufacturing centres versus office-based workplaces). Baseline sales data were also obtained from the 12-week period prior to the study (October to December 2021). Due to the timing of the study, there may have been some differences in food purchasing patterns given the on-going Covid-19 pandemic, although this was something that would have impacted all sites and should not impact the ability to measure the effectiveness of the intervention. Ethics approval was granted on 05/01/2022 by the Central University Research Ethics Committee, University of Oxford (Ref: R72710/RE004). The study adhered to CONSORT guidelines, and was pre-registered prospectively on ISRCTN (https://www.isrctn.com/ISRCTN10268258; 06/01/2022) and a Statistical Analysis Plan was pre-published on the Open Science Framework (https://osf.io/gr47t/; 03/03/2022).

The trial was initially designed to include three phases and three groups, with a step-wise introduction of ecolabels and increased relative availability of meat-free options. However, the planned increase in the availability of meat-free options was not implemented (Appendix A). As such, the trial was conducted with two groups during the primary intervention phase (Phase 1, lasting 6 weeks):

1. Control (no ecolabels).
2. Ecolabels added.

Following phase 1, there was a 2 week period with no change (Phase 2), after which ecolabels were introduced across all sites in Phase 3 (lasting 4 weeks).

### Randomisation

Randomisation was performed in Stata (Stata Statistical Software: Release 14. College Station, TX: StataCorp LP) by a statistician allocating a list of site names using random numbers. The catering provider identified 96 sites as eligible for the study. This was the maximum number of sites possible, as the trial aimed to be as large as was feasible. Sites were stratified based on mean daily transactions (< 120/day, 120–179, 180–249, 250–349, 350–599, 600–900, > 900). They were randomised to the originally planned study groups (control, ecolabels, availability). Given that availability was never increased, sites allocated to this group operated as control sites. When combined with the randomised-to-control group, together this group had approximately twice as many sites allocated to it as to the ecolabels condition. For the remainder of the manuscript, ‘control group’ refers to this combined grouping. We approached all sites to obtain contact details to allow fidelity checks and communication throughout the trial.

### Site identification and recruitment

This study included UK-based worksite cafeterias operated by a single nationwide catering provider. Inclusion criteria for sites were: having electronic point-of-sale tills operated by the catering provider, able to provide daily-level sales data that specified types and counts of meals sold, having a minimum of 50 transactions per day at baseline, and offering at least two main meal options, one of which was vegetarian, and which were selected from the base menu provided by the catering provider. All cafeterias operated a 4-week menu cycle, changing their main meals daily. Phone calls by the research team with site managers helped identify if there were any notable deviations from expected operation.

The catering provider recruited individual worksites according to the inclusion criteria and attained verbal consent from site managers. The sample size was based on pragmatic factors, primarily including the number of eligible sites identified that were likely to be operating (at least partially), since some were closed due to the Covid-19 pandemic. Cafeteria managers were asked not to draw attention to the research during the trial period.

Product-level daily sales data was provided by the catering provider for each site for the period 1st November 2021 to 17th April 2022. The trial started on the 24th of January, and all prior data served as baseline data for our analyses.

### Study procedures and interventions

#### Ecolabels

**Fig. 1:**
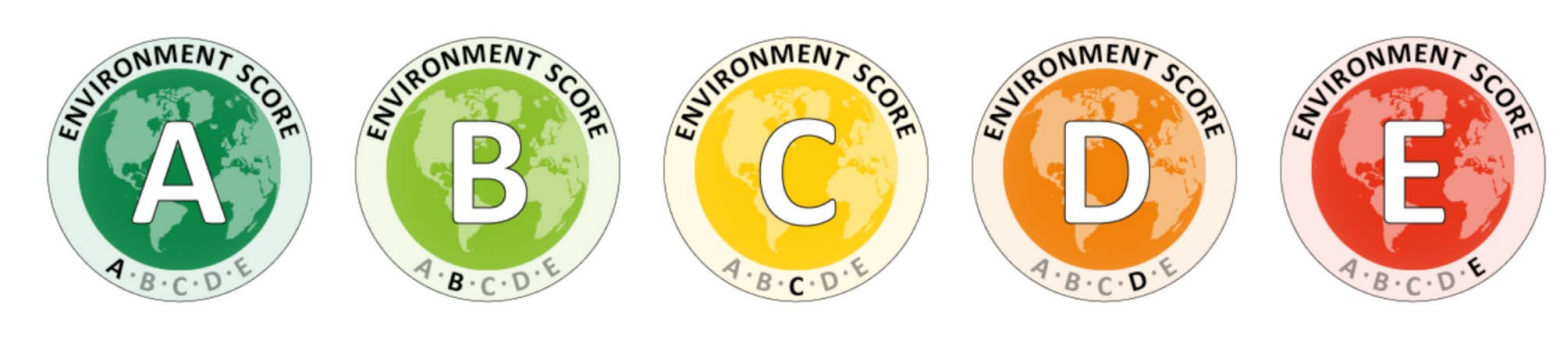
Ecolabels based on ingredient-level data obtained from the catering provider for each hot menu item sold. ‘**A**’ represents the lowest-impact, while ‘**E**’ represents the highest-impact

Menus and ingredient lists were provided by the catering provider, and we linked the ingredients in each recipe to an environmental database [17]. We calculated the environmental impacts of each meal, comprising four separate indicators - greenhouse gas emissions (GHGEs) (in kg CO_2_e), land-use related biodiversity loss (as species lost x 10^− 14^), scarcity-weighted water use (in litres), and eutrophication (in gPO_4_^3−^e) – and collapsed this into a single score, which could range from 0 to 100 and with each indicator equally weighted. For GHGEs and land-use related biodiversity loss, this weighting was done by dividing each indicator by the highest possible value for that indicator. This reflects a new scaling method for each indicator compared to previous research [12], but due to a technical error, water use and eutrophication were calculated as per a previous study [12], on the basis of rankings from highest to lowest impact. As a result, while products were generally in the same order for each indicator as if this technical error had not occurred, the impact score for how labels was assigned used a mix of the methodologies: with two indicator scores calculated as a percentage (from 0 to 100) of the worst possible value and two calculated as a result of ranking all possible items from 0 to 100, not accounting for the size of the difference between adjacent products. In all cases, values ranged from 0 to 100, with four scaled indicators ranked on a 0-100 scale. We then calculated environmental impact scores for each menu item in the hot meal categories (including jacket potatoes, noodle bar, hot savoury snacks (e.g., sausage rolls, pasties), sandwiches (e.g., toasties, panini), and soup), by taking the mean of the four indicators. Based on the single environmental impact score (range 0-100; where the lowest impact is better for the environment), meals with a score of e.g., 20 had 1/5th the overall impact of the highest scoring meal, whilst meals with a score of 50 had ½ the overall impact of the highest scoring meal. Meals were then categorised into A-E labels, such that meals scoring < 20 were given an ‘A’, 21–40 a ‘B’, 41–60 a ‘C’, 61–80 a D, and > 80 an E. Ecolabels (Fig. 1) displayed the ecolabel scores as one of five colour-coded (dark green to dark red) letters (A-E).

We designed these ecolabels following focus group sessions with members of the public, and have tested their effectiveness in a previous smaller field trial [12].

**Fig. 2:**
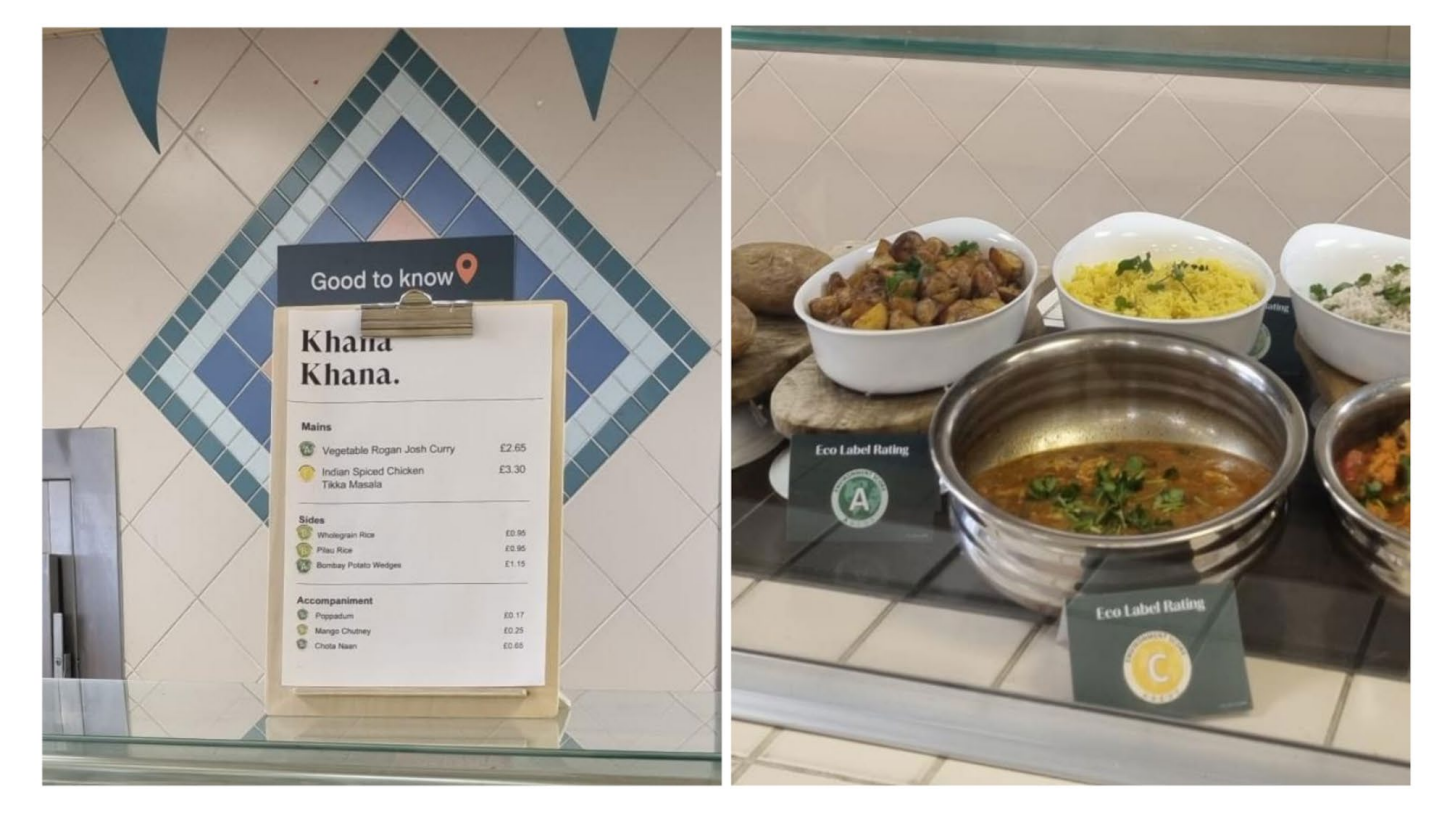
An example of ecolabels displayed on menus and ecolabel ‘tent cards’ positioned next to meal options on the hot counter

The catering provider uploaded the ecolabel A-E scores to their recipe software system, which could then be accessed and downloaded by site managers, and arranged for the printing and distribution of ecolabel ‘tent cards’ to intervention sites. Ecolabels appeared on menus next to meal options, and cafeteria staff were asked to position the ecolabel tent cards next to meal options on their hot meal counters (Fig. 2).

The catering provider also printed and displayed basic information sheets (see Supplementary Image 1) near the menus and elsewhere in the cafeteria to explain the ecolabel scoring.

### Fidelity checks

Due to the large number of sites located across the UK, together with Covid-19 pandemic restrictions, it was not possible to visit sites in person to assess the fidelity of the trial. Instead, members of the research team and trained community researchers had fortnightly calls with each site to check everything was running as planned and to resolve any queries. Site managers were asked to send weekly photos of their menus and hot meal counters to monitor the implementation of the interventions. Sites were ranked as having low, medium or high fidelity:

- Low - issues regarding labelling noted or only communicated/sent photos once or twice throughout the trial;
- Medium - no or only minor issues regarding labelling noted, but communication/photos were irregular;
- High - no or only minor issues regarding labelling noted, received regular communication/photos (i.e., more than 50% of requested photos received, no more than two consecutive gaps).

### Data preparation and analysis

#### Deviations from protocol

Due to delays in intervention implementation, Phase 1 of the study lasted for 6 weeks rather than 4 weeks, and Phase 2 was truncated to 2 weeks. The expected increase in meat-free availability was not observed (see supplementary section on Availability Manipulation Check in Appendix A), and so the trial group that was expected to have increased meat-free availability during Phase 1 was treated as part of the control group.

In terms of data, we were unable to obtain nutrition data as planned from the catering provider, which prevented us assessing the trial’s impact on nutritional outcomes. In addition, some imputation of environmental impact data was required (described below).

During our data checks, the technical error involving water use and eutrophication scores came to light. As a result, we used our planned outcome variable of total environmental impact, but we also examined the total label score (see Data Processing and Summary Variables), to account for any behavioural change in response to labels independent of change in exact environmental impact score.

### Data preparation

We removed data from bank holidays and weekends a priori, as not all sites were open. We also removed data from days in which sites informed us they were closed (e.g., due to refurbishments, extended Easter holidays, staff absences), which was ascertained during the fidelity check phone calls (via a set question).

#### Combining sales data with environmental impact data

We combined sales data recorded from point-of-sale tills with environmental impact data for each hot meal item. There were 831 unique hot meal items sold throughout the study, with 154 sold only during the baseline period, 126 sold only during the intervention periods, and 551 sold during both periods. Some imputation of environmental impact data was required (see Supplementary information). Examining sales quantities during the intervention period, 86% of items sold had ecolabel impact score values, we estimated impacts for 12% of meals sold, and we could not estimate impacts for 2% of meals sold. The catering provider’s base menu provided an A-E ecolabel score for all but these 2% of products to cafeteria staff.

### Data processing and summary variables

For each site and each trial phase, we examined the total number of items sold, total weekly revenue (£GBP), and used the environmental impact scores for each food product to calculate the total impacts per site from each individual environmental indicator (GHGEs, biodiversity, water use and eutrophication), and the total overall environmental impact. The total overall environmental impact per site was calculated by summing the environmental impacts from each hot meal item sold, and getting an aggregate score for the six-week Phase 1 period for each site. For each site and each trial phase, we also calculated the weekly environmental impacts of hot meal items in the same manner, by summing the ecolabel impact scores of all hot meal items sold during a given week. We also created a total label score, by summing the label values of all hot meal items sold, where A-E represented a score of 1–5 respectively. This outcome accounted for the behavioural response of cafeteria customers to the introduction of labels, given they were unaware of the underlying environmental impact scores.

### Statistical analysis

All analyses were conducted in Stata/IC Version 14.1. Analyses were intention-to-treat, unless otherwise specified. One sensitivity analysis was run on the primary outcome with per-protocol analysis. Assumptions of normality and constant variance were assessed, and outcome variables were log transformed for analyses.

#### Outcomes

The primary outcome was the total environmental impact per site from purchased hot meal items during each phase of the trial. Quantity of items sold was adjusted for in analysis models. We also examined the impacts on each of the four environmental indicators (GHGEs, biodiversity loss, water use, and eutrophication) separately.

Secondary outcomes assessed the impact of the intervention on total weekly revenue (£GBP), and the total number of items sold, for each site.

#### Primary analysis

Primary analyses used regression models, with a log-transformed outcome, to examine the impact of ecolabels on the total environmental impact, as well as on each of the four indicators (GHGEs, biodiversity loss, water use, and eutrophication), during Phase 1 of the trial. Predictors included in the models were whether ecolabels were present or not, total number of items sold and mean weekly baseline environmental impact of hot meal items. Significance was compared with an alpha of 0.05 for the primary analyses.

#### Secondary analysis

Secondary analyses used mixed effects modelling over the full 12 weeks of the trial to assess the impact of ecolabels. The models used the following predictors: group, total number of items sold, mean weekly baseline environmental impact of hot meal items, trial week and whether there was a bank holiday that week. Given the heterogeneous nature of the trial sites, we controlled for sites as a random effect. We modelled dummy variables for intention-to-treat with ecolabels. Significance was compared with an alpha of 0.016 for secondary analyses (Bonferroni adjustment).

#### Sensitivity analyses

As concerns were raised about fidelity in specific sites (e.g., there were often consecutive weeks when we were unable to communicate with site managers, many sites failed to send weekly photos, and some sites were known to be closed) we repeated our primary analysis excluding sites with low fidelity. We also conducted per-protocol analyses to adjust for the delays in implementation, where we coded intervention implementation variables to represent periods when the labels were actually implemented.

#### Exploratory analyses

We explored the percentage of meat vs. meat-free sales and availability over time by site grouping (Table 1), and the percentage of hot meals (a) on offer and (b) purchased, by ecolabel rating (i.e., A, B, C, D or E) over time by site grouping. We also explored the mean price of meat and meat-free hot meal items, calculated by including all products from the daily site offer.

#### Post-hoc analyses

The current study’s scoring method differed from the one used in our previous study [12], which had divided products into A-E based on proportional assignment (i.e., one fifth of menu products as an A, one fifth as a B, etc.), to better represent the scale of difference in environmental impacts. A post-hoc analysis re-analysed data from the previous study, applying the current method of environmental impact ranking, to compare baseline periods between studies, considering how environmental impact of menu items may have changed over time for the catering provider.

A final analysis was also run that used the total label score (‘A’ – ‘E’) as the outcome variable (see Data Processing and Summary Variables for how this was calculated), to assess the behavioural response of those in cafeterias. This used linear regression models to examine the impact of ecolabels on the total label score, which was comprised of the sum of all label values for all hot meal items sold in the given study period. After assessing for normality, this outcome was not log-transformed as was the case in the primary analysis models.

## Results

### Sample

**Fig. 3:**
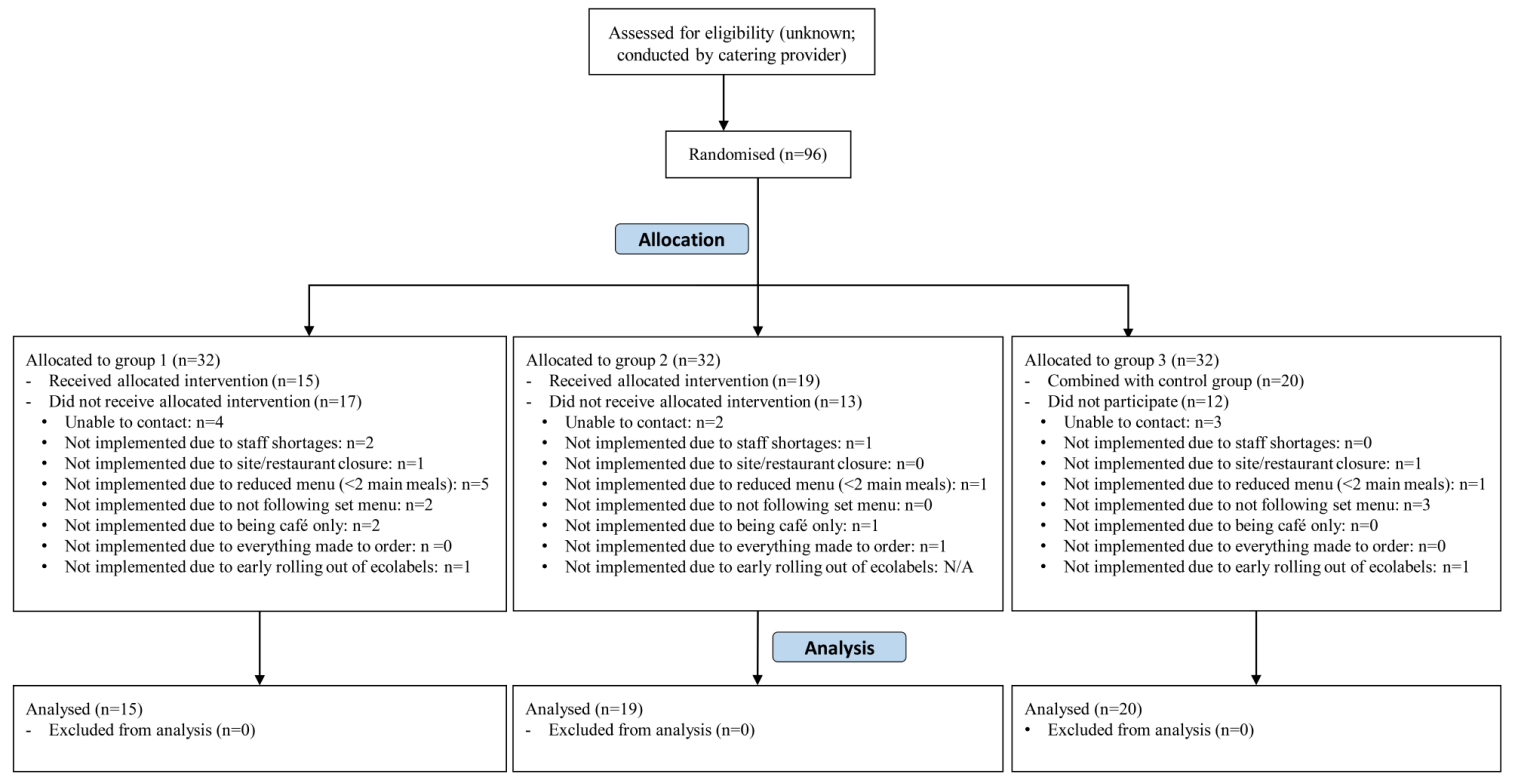
Flow diagram. Groups 1 and 3 were combined to form the control group; Group 2 is the ecolabel intervention group

Ninety-six sites across Great Britain were randomised, of which 54 (56%) completed the study, with 15 in the original control group (group 1; 28%), 19 in the ecolabel group (group 2; 35%) and 20 in availability group, which was later integrated into the control group (group 3; 37%) (Fig. 3).

Site dropouts occurred primarily due to difficulties in contacting site managers and sites withdrawing from the study due to their reporting being unable to meet the study requirements, such as through staff shortages. Some sites had temporary closures during the trial period, often due to staffing issues related to the Covid-19 pandemic. Additionally, some sites informed us they had deviated from the set menus, or offered reduced menus (with less than two main meals) due to the pandemic. In two cases, site managers implemented ecolabels earlier than planned, and had to be excluded since they were in the control group. All deviations from protocol were recorded by the study team during the fidelity checks and were accounted for during per-protocol analyses.

The mean weekly environmental impact of meals purchased during the baseline period was 5.40 in the combined control group 1 and 6.05 in the intervention group (on a scale of 0-100, reflecting a skew with fewer very high impact products and many lower impact products) (Table 1 for all descriptive statistics). See Supplementary Table 1 for mean values for the indicators which comprise the overall ecolabel impact score (i.e., GHGEs, water scarcity, land use related biodiversity loss, and eutrophication).

**Table 1:** Mean (SD) weekly number of hot meal items available and purchased, and mean weekly environmental impact score of items purchased in each trial phase by site grouping

	Mean weekly no. of meals available (SD)		Mean weekly no. of meals purchased (SD)		Mean weekly environmental impact score (SD)	
**Phase** **(with description)**	Control Group	Ecolabel Group	Control Group	Ecolabel Group	Control Group	Ecolabel Group
Baseline (no intervention)	52 (27)	54 (23)	370 (319)	705 (854)	5.40 (1.93)	6.05 (3.53)
Phase 1 (ecolabels applied in intervention group)	54 (30)	57 (26)	373 (390)	636 (739)	5.38 (1.71)	5.94 (3.32)
Phase 2 (no change)	53 (31)	57 (24)	387 (458)	615 (671)	5.63 (2.66)	5.85 (3.57)
Phase 3 (all groups apply ecolabels)	48 (21)	51 (20)	359 (239)	554 (576)	5.20 (1.67)	5.76 (3.20)

During the baseline period, the mean price of meat-free hot meal items was £1.82 (SD: £0.93), and of meat items was £2.40 (SD: £0.79). During the trial period, the mean price of meat-free hot meal items was £1.81 (SD: £0.96), and the mean price of all meat items was £2.40 (SD: £0.79).

### Effect of ecolabels on environmental impact

There was no significant difference in the change in the total environmental impact of purchased hot meal items between control sites and intervention sites during the 6 weeks where ecolabels were present (1.4% decrease, 95%CI: -33.6%, 30.8%). A per-protocol regression model for total environmental impact (-2.9%, 95%CI: -30.2%, 35.2%) and the mixed effects model, which analysed the total environmental impact using total weekly impacts over the full 12 weeks, also showed no effect (-5.4%, 95%CI: -12.6%, 2.5%).

A sensitivity analysis, which excluded sites with low fidelity (*N* = 32, with 22 excluded), also found no difference in the change in total environmental impact between sites with ecolabels and without (10.8% decrease, 95%CI: -45.3%, 45.5%). Similarly, the post-hoc analysis on total label score (i.e. where A = 1, B = 2, etc.), which explored any effect on behaviour independent of specific product environmental scores, also found no significant impact of adding labels to menus, with a change in total label score of -96 (95%CI: -283, 91).

No effect of ecolabels was observed for the individual environmental indicators (GHGEs: -3.6%, 95%CI: -30.7%, 34.3%; biodiversity loss: 2.0%, 95%CI: -25.8%, 40.2%; eutrophication: -2.4%, 95%CI: -29.3%, 34.7%; water scarcity: -0.4%, 95%CI: -28.7%, 39.1%). For full regression and mixed model outputs see Supplementary Tables 4 & 5.

**Fig. 4:**
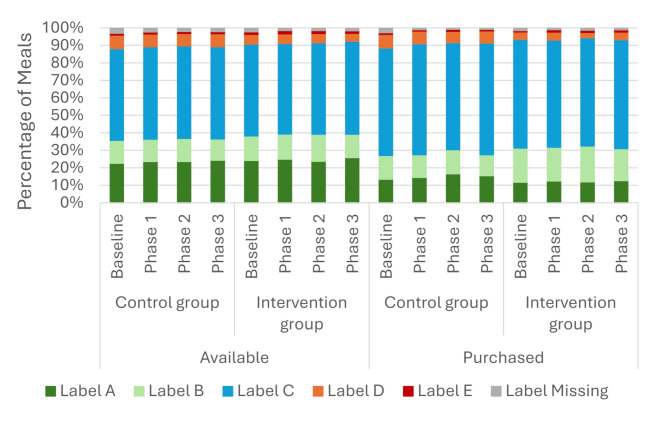
Percentage (%) of labelled hot main meals available and purchased, by ecolabel rating over time by site grouping. No score indicates those items such as “SOUP 12OZ”, which were purchased but for which no ecolabel score data was available

In both groups and all trial phases, the majority of hot meal items available had an ecolabel rating of C, while around a quarter were rated A. Less than 2% of hot meal items had an E rating. This pattern was reflected in the meals purchased (Fig. 4).

### Additional outcomes

*Revenue*: Mixed models showed a decrease in weekly revenue (£GBP) in sites that had ecolabels present compared to control (-4.0% decrease in revenue, 95%CI: -1.2%, -6.7%), but no change in total items sold (0.3% increase in number of items sold, 95%CI: -6.1%, 5.2%).

*Meals offered*: A post-hoc analysis of the baseline data from our research team’s previous study [12], compared with baseline data in this study, found no difference in mean environmental impact of items offered (all hot meal items labelled/scored: +0.096 change in impact score, 95%CI: -0.50, 0.69; meals only: +0.01 change, 95%CI: -0.11, 0.12).

#### Cafeteria manager feedback

Through calls to sites, which provided informal feedback on how the intervention was going, some managers cited concerns over food waste, low customer numbers likely due to the COVID-19 pandemic situation, and customer preference for meat options as reasons they did not offer more meat-free options.

## Discussion

This study set out to investigate the effectiveness of two strategies to nudge consumers towards more environmentally sustainable meal options in UK-based worksite cafeterias - ecolabels, and increased meat-free meal availability – individually and in combination. Availability was not increased and eco-labels were ineffective in changing consumer demand. There was no change in the number of meals sold, but mean weekly revenue (£GBP) decreased when ecolabels were present, and meat-free options, on average, cost less. We hypothesised that increasing availability may help increase the effectiveness of ecolabels, but this could not be tested.

This large, canteen-based study found no evidence of an effect of ecolabels, similar to our previous study in 28 worksite cafeterias [12]. Nor was there any evidence that ecolabels prompted a change in industry behaviour, through changes in the supply of food for purchase. At a head office level, there was some effort to change the array of meals available, however this resulted in minimal changes in environmental impact of actual menus offered, where site managers were able to express a choice of items to offer in their individual settings. This meant we were unable to test the effectiveness of increasing meat-free availability, and also means that additional work is needed to understand how to appropriately incentivise site managers to effectively shift meal availability towards more sustainable options. Other outcomes such as revenue and total number of items sold were assessed in exploratory analyses to better understand broader consumer purchasing behaviour in the cafeterias at this time. Many factors could influence a change in revenue, including that meat-free options tended to be sold at a lower price, however, given the evidence available in this study, it is not possible to determine why any change may have occurred.

One of the main strengths of this study was its real-world setting and size, with 54 worksites from across Britain, inclusive of both manufacturing, factory, and office workforces. Regular communication with site managers as well as the collection of weekly photos provided valuable insights into the implementation of ecolabels and any associated challenges. However, there were also limitations. This study was conducted over a relatively short period of time (i.e., 6 weeks for the primary RCT component), and this may have limited our ability to detect small, but potentially important changes. Further, allocation to study groups was originally done based on an expected sample of 96 sites and three study groups, so uneven allocation may have lowered the power and the ability to pick up on small effects. It also took place during the Covid-19 pandemic and we were not able to visit any sites in person. Several sites also reported worker shortages and reduced customer traffic. These circumstances affected the implementation of ecolabels as there were instances where cafeteria staff were unable to label all products due to time constraints. While these conditions posed difficulties, they also provided insights into the practical limitations and barriers that may impact the implementation or performance of ecolabels in a realistic setting.

Another limitation is the restricted range of ecolabel scores of the food available. On most days, there were a predominance of hot meal items rated ‘C’, followed by ‘A’ and ‘B’. While any shift from the ‘C’ meal to a lower meal may have had the potential to meaningfully contribute to a reduction in the environmental impact of food consumed at a given site, however, given that many meals were rated ‘C’ there was likely less opportunity for more substantial shifts to occur. Previous experimental studies have shown that highlighting the worst option or providing a range of options tends to have a greater impact, particularly in cases where a choice has societal or public impacts [18]. This suggests individuals may be more inclined to move away from a worse option rather than towards the best option. Both studies run by our research group have resulted in a limited capacity for consumers to shift behaviour between ecolabel scores, restricting the potential for ecolabels to influence behaviour. This is particularly important to consider in the context of implementing ecolabels as part of a policy option. There have been a variety of methods by which sustainable food profiling models score foods, with no international standard [19], and the differences in the range of scores found within these profiling models may be of particular relevance when considering their impact. Our research findings illustrate that additional thought needs to be put into ecolabels to consider whether there is a need (a) for a variety of ecolabel values for different types of dishes (mains, sides, desserts, etc.), and (b) to understand how using different cut-off scores for the different ecolabel values may most effectively shift behaviours towards more sustainable food choices.

In worksite cafeterias, where decisions are made quickly and habits play a significant role, limited meat-free options and customer preferences for meat options can impact purchasing behaviours. Site managers reported low customer interest in meat-free options, resulting in potentially limited selection of these items from base menus to minimise food waste. This may also have impacted our assessment of availability: if availability increased, but no one purchased these items, these items would not appear in the dataset, and would not be recorded as being available. However, calls with site managers did not suggest this occurred. Coinciding with this, but likely unrelated to our study, there was also a general reduction in footfall at these worksites with the ongoing Covid-19 pandemic. Site managers were having to estimate how much food they would need to make while still trying to limit food waste (i.e. not make more main meal options than needed on a given day). If site managers were particularly concerned about customers’ response to meat-free options, this may have played a role in the lack of increase in these options in the availability condition, and raises questions about how meat-free availability could be increased without the potential for knock-on effects of customers going elsewhere to purchase the foods they may prefer consuming (e.g., meat hot meal items). Future trials should consider adopting a more direct one-to-one approach, working directly with site managers rather than through central communication to encourage adoption of the intervention. This would enable a more personalised approach to address potential barriers, including concerns over the backlash they may face from their sites, or to incentivise adoption. There also may be some benefit in considering how to support cafeterias to use language or imagery around vegetarian options to improve their uptake, as studies have found this to be a successful intervention, and build confidence among site managers that these interventions would be popular [20, 21].

Our findings can be contextualized with literature on other labelling interventions, such as calorie labelling. While meta analyses have suggested that calorie labels have an effect on both consumer selections and catering outlet offerings [22], it has been suggested that the effect of these labelling initiatives may be so small that both time, which can allow small downward trends to become more apparent, as well as very large high-powered studies may be required for individual studies to detect any changes occurring [23]. As such, while this study found no evidence that ecolabels were effective at changing consumer behaviour, it is possible that there may be a smaller impact than could be detected in the current study, or that impact may develop over time. In addition, implementation of ecolabels could potentially change the behaviour of catering providers as well as consumers; however, it is not yet possible to fully test this.

## Conclusion

In our study, conducted during the Covid-19 pandemic, we found no evidence that adding ecolabels to hot menu items in worksite canteens changed purchasing behaviours, or altered the meals which were offered on menus.

## Electronic supplementary material

Below is the link to the electronic supplementary material.

Supplementary Material 1

Supplementary Material 2

## Data Availability

The dataset comprises commercially sensitive data so is not publicly available, but are available from the corresponding author on reasonable request and with the permission of the catering provider.
