# Supplementary material for: Testing the effect of ecolabels on the environmental impact of food purchases in worksite cafeterias: a randomised controlled trial
Source: BMC Public Health. 2025 Jan 11;25:127. doi: 10.1186/s12889-024-21272-4 (PMC11724574; doi:10.1186/s12889-024-21272-4)

**Supplementary Materials: Testing the effect of ecolabels on the environmental impact of food purchases in worksite cafeterias: A randomised controlled trial**

**Supplementary Information A**. Trial including a planned availability increase

The trial was originally planned to have three phases. Cafeterias were allocated to one of three study groups for a period of 6 weeks (‘Phase 1’):

- group 1: control (neither ecolabels nor increased meat-free availability),
- group 2: ecolabels only,
- group 3: increased meat-free availability only.

Following Phase 1, increased meat-free availability and ecolabels were to be introduced across the sites in a stepwise manner. In Phase 2, lasting 2 weeks, meat-free availability was planned to increase across all sites. All phases were originally planned for 4 weeks, but due to implementation delays at some sites, Phase 1 was extended to run for 6 weeks and Phase 2 was shortened to 2 weeks. In Phase 3, ecolabels were introduced in the remaining groups, so that all sites had both ecolabels and increased meat-free availability for the final 4-week period (Appendix A Table 1).

**Supplementary Appendix A Table 1**. Study Design

| Phase | Weeks | Group 1  (control) | Group 2  (ecolabels) | Group 3  (increased availability of meat-free options) |
| --- | --- | --- | --- | --- |
| 1 | 1-6 | - No ecolabels  - No increased meat-free availability | + Ecolabels  - No increased meat-free availability | - No ecolabels  + Increased meat-free availability |
| 2 | 7-8 | - No ecolabels  + Increased meat-free availability | + Ecolabels  + Increased meat-free availability | - No ecolabels  + Increased meat-free availability |
| 3 | 9-12 | + Ecolabels  + Increased meat-free availability | + Ecolabels  + Increased meat-free availability | + Ecolabels  + Increased meat-free availability |

Plans to Increase availability

The catering provider used a base menu, which consisted of all the potential meal options a site could serve. Individual site managers chose meals to serve in their sites from the base menu, rather than offering this menu in full. The planned increase in availability of meat-free hot meal items (forming part of this trial, but also as part of the catering provider’s sustainability strategy), was operationalised by producing a new base menu, which included an increased proportion of meat-free options. This meant, in Phase 1, the increased availability group had the new base menu from which to choose their hot meal options, and the other two groups had the older base menu to choose from. In all Phases, the actual availability of meat-free options in the worksite cafeterias was determined by the hot meal items site managers selected for their individual site menus.

Meat-free availability manipulation check

The proportions of meat-free main meal options available and purchased over time and by site grouping show some fluctuations, however they do not differ over time or by group in accordance with the planned availability increases (Appendix A Table 2). Analysis also showed that changes on the base menus for sites to choose from were minimal, with only a few additional lower impact options on the menus (See Supplementary Table 2 & 3).

**Appendix A Table 2**. Mean weekly percentage (%) of meat-free meals available and purchased over time by site grouping. Shading shows where availability was planned to be increased, with variability in where or if it was increased, irrespective of shaded area. Availability was estimated from sales data.

|  | **Mean Meat-free availability (SD)** | | | **Mean Meat-free purchases (SD)** | | |
| --- | --- | --- | --- | --- | --- | --- |
| **Phase** | Group 1 | Group 2 | Group 3 | Group 1 | Group 2 | Group 3 |
| Baseline | 37.6 (10.0) | 37.1 (10.0) | 36.7 (12.2) | 26.9 (12.2) | 24.1 (8.6) | 24.1 (10.6) |
| 1 | 39.4 (11.6) | 36.5 (9.6) | 38.2 (11.6) | 26.4 (12.2) | 23.4 (8.0) | 24.8 (10.9) |
| 2 | 40.5 (10.5) | 35.1 (9.3) | 38.1 (12.1) | 30.7 (15.2) | 21.2 (7.8) | 26.4 (12.3) |
| 3 | 37.0 (10.6) | 35.5 (11.2) | 39.1 (11.3) | 23.9 (11.2) | 21.4 (8.1) | 24.6 (10.6) |

If a site offered at least two additional meat-free options per week as compared with the baseline period, we deemed meat-free meal availability to have increased. In Phase 1, where increased meat-free availability was planned for Group 3, thirteen sites (24%) across all three groups met the criteria for availability increase (five sites (33%) in Group 1, three sites in Group 2 (15.8%), and five sites (25%) in Group 3). Given increased availability was not seen in Group 3 relative to the other trial groups, this group was treated as akin to the control, having not applied ecolabels (analyses were also run treating these as three groups, and did not change results).

This further emphasises some of the challenges with implementing these studies or with considering this for a real intervention. Worksite cafeterias have control over which options from the base menu they select for their own menus, so if the foods they happen to select for that cycle tend to be more meat-free options, then this could happen even without being told to do so.

**Supplementary Information B**. Imputation methods for missing product environmental impact scores

The meals with imputed impacts comprised 329 products of the 831 unique hot meal items on the base menu (40% of unique meals; with 258 sold during baseline and 217 sold during intervention periods). These were missing the underlying environmental impact data (i.e., the four individual environmental impact values and mean environmental impact score). We had analysed these menu items previously for other events run by the catering provider in previous years, and as such, an A-E label was attached to the hot meal item. Due to a technical issue, we were unable to retrieve the full recipes and full environmental impact data for these products. To impute these missing data, we used a two-step approach. First, we matched items on A-E ecolabel score and similar product titles with products we did have environmental data for (e.g., matching ‘Soup parsnip spiced fresh’ with ‘soup parsnip spiced’); we were able to do this for 75 products (9% of the 831 products, 3.7% of all products sold). For the remaining products (n=237, 29% of the 831 products, 12% of all products sold), where possible, we imputed missing data with average values calculated across the same product category (e.g., hot sandwiches) and A-E ecolabel score (e.g., B). Following this, 17 items (2%) still had no ecolabel score, but these were generically named items (e.g., “Soup 12OZ”) and could not be identified. These were the same 2% of products missing an ecolabel value. Ultimately, 98% of items sold during the intervention period had environmental impact data for analysis.

Supplementary Table 1. Mean weekly ecoscore, and total greenhouse gas emissions, eutrophication, biodiversity loss, and water scarcity of items purchased in each trial phase by site grouping.

|  |  | Study Period | Mean | Std. Dev. | Min | Max |
| --- | --- | --- | --- | --- | --- | --- |
| Group 1 | Total ecoscore (from weekly aggregate of food scores) | Baseline | 5.03 | 1.50 | 3.23 | 8.90 |
|  |  | Phase 1 | 4.84 | 1.10 | 3.55 | 6.97 |
|  |  | Phase 2 | 5.25 | 2.10 | 3.01 | 11.00 |
|  |  | Phase 3 | 5.20 | 1.67 | 3.21 | 9.67 |
|  | Greenhouse gas emissions (GHGEs) (kg CO_2_e) | Baseline | 5106 | 2722 | 786 | 9885 |
|  |  | Phase 1 | 2460 | 1328 | 375 | 4840 |
|  |  | Phase 2 | 816 | 492 | 113 | 1676 |
|  |  | Phase 3 | 1570 | 904 | 229 | 3007 |
|  | Aquatic eutrophication (gPO_4_^3-^e) | Baseline | 21060 | 10667 | 3116 | 38260 |
|  |  | Phase 1 | 9999 | 5520 | 1591 | 21614 |
|  |  | Phase 2 | 3365 | 2024 | 517 | 7488 |
|  |  | Phase 3 | 6594 | 4005 | 949 | 13793 |
|  | Land use related biodiversity loss (species lost x  10^-14^) | Baseline | 45614 | 23571 | 7627 | 86947 |
|  |  | Phase 1 | 21933 | 12577 | 5126 | 49554 |
|  |  | Phase 2 | 7469 | 4671 | 1381 | 17190 |
|  |  | Phase 3 | 14839 | 9277 | 2301 | 30214 |
|  | Scarcity weighted freshwater withdrawals (litres) | Baseline | 11800000 | 6617732 | 1961356 | 23100000 |
|  |  | Phase 1 | 5829142 | 3319636 | 982776 | 12900000 |
|  |  | Phase 2 | 2014903 | 1419766 | 269145 | 5553627 |
|  |  | Phase 3 | 3828861 | 2371541 | 533094 | 9390523 |
| Group 2 | Total ecoscore (from weekly aggregate of food scores) | Baseline | 6.05 | 3.53 | 1.94 | 19.32 |
|  |  | Phase 1 | 5.94 | 3.32 | 2.38 | 18.23 |
|  |  | Phase 2 | 5.85 | 3.57 | 2.03 | 19.25 |
|  |  | Phase 3 | 5.76 | 3.20 | 2.13 | 16.91 |
|  | Greenhouse gas emissions (GHGEs) (kg CO_2_e) | Baseline | 9709 | 9281 | 1518 | 33102 |
|  |  | Phase 1 | 4716 | 4551 | 516 | 16075 |
|  |  | Phase 2 | 1422 | 1197 | 194 | 4535 |
|  |  | Phase 3 | 2569 | 2239 | 455 | 7360 |
|  | Aquatic eutrophication (gPO_4_^3-^e) | Baseline | 38998 | 36470 | 5674 | 132366 |
|  |  | Phase 1 | 18981 | 18064 | 2469 | 61456 |
|  |  | Phase 2 | 5777 | 4923 | 893 | 17416 |
|  |  | Phase 3 | 10482 | 9332 | 1826 | 29537 |
|  | Land use related biodiversity loss (species lost x  10^-14^) | Baseline | 90708 | 93695 | 9738 | 304838 |
|  |  | Phase 1 | 45116 | 46272 | 6198 | 155301 |
|  |  | Phase 2 | 13956 | 12947 | 2235 | 44925 |
|  |  | Phase 3 | 25256 | 23912 | 3977 | 77608 |
|  | Scarcity weighted freshwater withdrawals (litres) | Baseline | 22500000 | 20400000 | 3209909 | 68500000 |
|  |  | Phase 1 | 12000000 | 11700000 | 1582717 | 38500000 |
|  |  | Phase 2 | 3686864 | 3569516 | 543089 | 13400000 |
|  |  | Phase 3 | 6659835 | 6456446 | 1190841 | 24100000 |
| Group 3 | Total ecoscore (from weekly aggregate of food scores) | Baseline | 5.68 | 2.16 | 2.40 | 12.24 |
|  |  | Phase 1 | 5.79 | 1.96 | 3.22 | 9.24 |
|  |  | Phase 2 | 5.91 | 2.99 | 2.86 | 14.81 |
|  |  | Phase 3 | 5.59 | 2.13 | 2.37 | 12.28 |
|  | Greenhouse gas emissions (GHGEs) (kg CO_2_e) | Baseline | 6048 | 5891 | 662 | 22412 |
|  |  | Phase 1 | 3139 | 3302 | 185 | 12631 |
|  |  | Phase 2 | 1092 | 1223 | 137 | 4445 |
|  |  | Phase 3 | 2030 | 2597 | 261 | 10732 |
|  | Aquatic eutrophication (gPO_4_^3-^e) | Baseline | 24431 | 23083 | 3045 | 89575 |
|  |  | Phase 1 | 12597 | 13115 | 716 | 51385 |
|  |  | Phase 2 | 4445 | 5059 | 484 | 18534 |
|  |  | Phase 3 | 8180 | 10506 | 1068 | 43670 |
|  | Land use related biodiversity loss (species lost x  10^-14^) | Baseline | 49483 | 41294 | 6605 | 158539 |
|  |  | Phase 1 | 25959 | 24294 | 1518 | 91282 |
|  |  | Phase 2 | 9166 | 9976 | 957 | 34363 |
|  |  | Phase 3 | 16857 | 18968 | 2097 | 75242 |
|  | Scarcity weighted freshwater withdrawals (litres) | Baseline | 14900000 | 15100000 | 1297168 | 56800000 |
|  |  | Phase 1 | 7944745 | 8511505 | 385434 | 33000000 |
|  |  | Phase 2 | 2946255 | 3775518 | 176292 | 12300000 |
|  |  | Phase 3 | 5342474 | 7303375 | 381854 | 28800000 |
| Group 1 & 3 (control in analyses) | Total ecoscore (from weekly aggregate of food scores) | Baseline | 5.40 | 1.93 | 2.40 | 12.24 |
|  |  | Phase 1 | 5.38 | 1.71 | 3.22 | 9.24 |
|  |  | Phase 2 | 5.63 | 2.66 | 2.86 | 14.81 |
|  |  | Phase 3 | 5.42 | 1.95 | 2.37 | 12.28 |
|  | Greenhouse gas emissions (GHGEs) (kg CO_2_e) | Baseline | 5644 | 4805 | 662 | 22412 |
|  |  | Phase 1 | 2848 | 2657 | 185 | 12631 |
|  |  | Phase 2 | 974 | 986 | 113 | 4445 |
|  |  | Phase 3 | 1833 | 2057 | 229 | 10732 |
|  | Aquatic eutrophication (gPO_4_^3-^e) | Baseline | 22987 | 18813 | 3047 | 89575 |
|  |  | Phase 1 | 11483 | 10600 | 716 | 51385 |
|  |  | Phase 2 | 3982 | 4071 | 484 | 18534 |
|  |  | Phase 3 | 7500 | 8376 | 949 | 43670 |
|  | Land use related biodiversity loss (species lost x  10^-14^) | Baseline | 47825 | 34765 | 6610 | 158539 |
|  |  | Phase 1 | 24234 | 20164 | 1518 | 91282 |
|  |  | Phase 2 | 8439 | 8157 | 957 | 34362 |
|  |  | Phase 3 | 15992 | 15557 | 2097 | 75242 |
|  | Scarcity weighted freshwater withdrawals (litres) | Baseline | 13500000 | 12300000 | 1297167 | 56800000 |
|  |  | Phase 1 | 7038069 | 6852412 | 385434 | 33000000 |
|  |  | Phase 2 | 2547060 | 3028336 | 176276 | 12300000 |
|  |  | Phase 3 | 4693776 | 5767777 | 381854 | 28800000 |

Supplementary Table 2. Base weekly menus showing the percentage of options with each label that were available for catering managers to select, with the planned increased availability given to sites in Group 2, and the menu without planned availability increase given to Groups 1 and 3.

| Weekly Menu (Menu with planned increased availability of meat-free) | | | | |
| --- | --- | --- | --- | --- |
|  | Week 1 | Week 2 | Week 3 | Week 4 |
| A | 41.2% | 33.8% | 35.4% | 34.7% |
| B | 26.5% | 36.4% | 31.8% | 31.4% |
| C | 30.2% | 25.0% | 28.2% | 29.2% |
| D | 2.1% | 2.2% | 4.7% | 4.8% |
| E | 0.0% | 2.6% | 0.0% | 0.0% |
| Weekly Menu (Menu without planned meat-free availability increase) | | | | |
|  | Percent Week 1 | Percent Week 2 | Percent Week 3 | Percent Week 4 |
| A | 41.0% | 32.8% | 38.7% | 38.1% |
| B | 26.6% | 36.9% | 30.1% | 29.7% |
| C | 30.3% | 25.4% | 26.7% | 27.6% |
| D | 2.1% | 2.2% | 4.5% | 4.6% |
| E | 0.0% | 2.6% | 0.0% | 0.0% |

Supplementary Table 3. Base menu options showing the percentage of options with each label that were available for catering managers to select, with the planned increased availability given to sites in Group 2, and the menu without planned availability increase given to Groups 1 and 3.

| Menu Items (Menu with planned increased availability of meat-free) | | | | | | | | | | | |
| --- | --- | --- | --- | --- | --- | --- | --- | --- | --- | --- | --- |
|  | Premade-Core | FTG Breakfast | Full Hot Breakfast | Toast Station | Porridge Station | Fruit, Yog & Cereal Bar | Pastries | Soup Station | Jacket Potato Station | Grab & Go | Bakery |
| A | 4.1% | 2.7% | 50.0% | 68.0% | 45.0% | 64.7% | 62.5% | 80.0% | 28.6% | 29.9% | 45.5% |
| B | 38.8% | 29.7% | 15.9% | 32.0% | 50.0% | 29.4% | 0.0% | 20.0% | 33.3% | 50.6% | 31.8% |
| C | 56.1% | 67.6% | 34.1% | 0.0% | 5.0% | 5.9% | 37.5% | 0.0% | 23.8% | 19.5% | 22.7% |
| D | 1.0% | 0.0% | 0.0% | 0.0% | 0.0% | 0.0% | 0.0% | 0.0% | 14.3% | 0.0% | 0.0% |
| E | 0.0% | 0.0% | 0.0% | 0.0% | 0.0% | 0.0% | 0.0% | 0.0% | 0.0% | 0.0% | 0.0% |
| Menu Items (Menu without planned increase of meat-free availability) | | | | | | | | | | | |
|  | Premade-Core- | FTG Breakfast | Full Hot Breakfast | Toast Station | Porridge Station | Fruit, Yog & Cereal Bar | Pastries | Soup Station | Jacket Potato Station | Grab & Go | Bakery |
| A | 4.1% | 2.7% | 48.8% | 68.0% | 45.0% | 64.7% | 62.5% | 80.0% | 28.6% | 31.3% | 45.5% |
| B | 38.8% | 29.7% | 16.3% | 32.0% | 50.0% | 29.4% | 0.0% | 20.0% | 33.3% | 50.6% | 31.8% |
| C | 56.1% | 67.6% | 34.9% | 0.0% | 5.0% | 5.9% | 37.5% | 0.0% | 23.8% | 18.1% | 22.7% |
| D | 1.0% | 0.0% | 0.0% | 0.0% | 0.0% | 0.0% | 0.0% | 0.0% | 14.3% | 0.0% | 0.0% |
| E | 0.0% | 0.0% | 0.0% | 0.0% | 0.0% | 0.0% | 0.0% | 0.0% | 0.0% | 0.0% | 0.0% |

Supplementary Table 4. Full linear regression outputs, including sensitivity analyses and secondary outcomes. Note: most outcome variables are a log-transformed outcome, except the post-hoc analysis.

| Primary analysis: log-transformed outcome of site total impact in Phase 1 (N=54) | | | | | | |
| --- | --- | --- | --- | --- | --- | --- |
|  | Coef. | Std. Err. | t | P-value | 95% CI | |
| Ecolabels present (intention-to-treat) (Reference: no ecolabels) | -0.014 | 0.16 | -0.09 | 0.931 | -0.34 | 0.31 |
| Quantity of item sold | 0.0002 | 0.00 | 9.79 | <0.001 | 0.0002 | 0.0003 |
| Total impact in the baseline period | 13.83 | 2.89 | 4.79 | <0.001 | 8.04 | 19.63 |
| Constant | 3.19 | 0.20 | 16.29 | <0.001 | 2.80 | 3.59 |
| Sensitivity analysis: Log outcome of site total impact in Phase 1, per-protocol analysis (N = 54) | | | | | | |
|  | Coef. | Std. Err. | t | P-value | 95% CI | |
| Ecolabels present (per-protocol) (Reference: no ecolabels) | -0.029 | 0.17 | -0.18 | 0.860 | -0.36 | 0.30 |
| Quantity of item sold | 0.001 | 0.0001 | 9.59 | <0.001 | 0.001 | 0.002 |
| Mean weekly impact in baseline period | 13.80 | 2.91 | 4.74 | <0.001 | 7.95 | 19.66 |
| Constant | 1.40 | 0.20 | 7.1 | <0.001 | 1.01 | 1.80 |
| Sensitivity analysis: Log outcome of site impact in Phase 1, sensitivity analysis for site fidelity (N = 32) | | | | | | |
|  | Coef. | Std. Err. | t | P-value | 95% CI | |
| Ecolabels present (intention-to-treat) (Reference: no ecolabels) | -0.11 | 0.24 | -0.48 | 0.636 | -0.60 | 0.38 |
| Quantity of item sold | 0.0002 | 0.00004 | 6.03 | <0.001 | 0.0002 | 0.0003 |
| Mean weekly impact in baseline period | 12.21 | 3.82 | 3.19 | 0.003 | 4.38 | 20.04 |
| Constant | 3.33 | 0.27 | 12.34 | <0.001 | 2.77 | 3.88 |
| Secondary outcome: Log outcome of site GHGEs in Phase 1 (N=54) | | | | | | |
|  | Coef. | Std. Err. | t | P-value | 95% CI | |
| Ecolabels present (intention-to-treat) (Reference: no ecolabels) | -0.036 | 0.16 | -0.22 | 0.827 | -0.37 | 0.30 |
| Quantity of item sold | 0.0002 | 0.00002 | 9.58 | <0.001 | 0.0002 | 0.0003 |
| Mean weekly impact in baseline period | 13.45 | 2.97 | 4.53 | <0.001 | 7.49 | 19.42 |
| Constant | 6.34 | 0.20 | 31.40 | <0.001 | 5.93 | 6.74 |
| Secondary outcome: Log outcome of site biodiversity loss impact in Phase 1 (N=54) | | | | | | |
|  | Coef. | Std. Err. | t | P-value | 95% CI | |
| Ecolabels present (intention-to-treat) (Reference: no ecolabels) | 0.020 | 0.16 | 0.12 | 0.901 | -0.30 | 0.34 |
| Quantity of item sold | 0.0002 | 0.00002 | 9.87 | <0.001 | 0.0002 | 0.0003 |
| Mean weekly impact in baseline period | 12.60 | 2.85 | 4.42 | <0.001 | 6.87 | 18.33 |
| Constant | 8.57 | 0.19 | 44.24 | <0.001 | 8.18 | 8.96 |
| Secondary outcome: Log outcome of site aquatic eutrophication impact in Phase 1 (N=54) | | | | | | |
|  | Coef. | Std. Err. | t | P-value | 95% CI | |
| Ecolabels present (intention-to-treat) (Reference: no ecolabels) | -0.025 | 0.161 | -0.15 | 0.879 | -0.35 | 0.30 |
| Quantity of item sold | 0.0002 | 0.00002 | 9.71 | <0.001 | 0.0002 | 0.0003 |
| Mean weekly impact in baseline period | 13.57 | 2.89 | 4.69 | <0.001 | 7.76 | 19.38 |
| Constant | 7.74 | 0.20 | 39.37 | <0.001 | 7.34 | 8.13 |
| Secondary outcome: Log outcome of site water scarcity impact in Phase 1 (N=54) | | | | | | |
|  | Coef. | Std. Err. | t | P-value | 95% CI | |
| Ecolabels present (intention-to-treat) (Reference: no ecolabels) | -0.004 | 0.166 | -0.020 | 0.982 | -0.34 | 0.33 |
| Quantity of item sold | 0.0002 | 0.00002 | 9.46 | <0.001 | 0.0002 | 0.0003 |
| Mean weekly impact in baseline period | 15.95 | 3.00 | 5.33 | <0.001 | 9.93 | 21.97 |
| Constant | 13.99 | 0.20 | 68.74 | <0.001 | 13.58 | 14.40 |
| Post-hoc analysis outcome: total label score in Phase 1 (N=54) | | | | | | |
|  | Coef. | Std. Err. | t | P>t | 95% CI | |
| Ecolabels present (intention-to-treat) (Reference: no ecolabels) | -95.8 | 93.2 | -1.0 | 0.309 | -282.9 | 91.3 |
| Quantity of item sold | 2.6 | 0.01 | 195.9 | <0.001 | 2.6 | 2.6 |
| Total impact in the baseline period | 2118.4 | 182.9 | 11.6 | <0.001 | 1751.0 | 2485.9 |
| Constant | -5333.9 | 474.4 | -11.2 | <0.001 | -6286.8 | -4380.9 |

Supplementary Table 5. Mixed model outputs for environmental impact, revenue, quantity. Coefficients in tables are for fixed effects, random effects were the worksite cafeteria locations.

| Secondary analysis: Mixed model for log-transformed weekly impact by site (N=54) | | | | | | |
| --- | --- | --- | --- | --- | --- | --- |
|  | Coef. | Std. Err. | z | P-value | [95% Conf. | Interval] |
| Ecolabels present (intention-to-treat) | -0.06 | 0.04 | -1.36 | 0.174 | -0.13 | 0.02 |
| Total Quantity | 0.001 | 0.0001 | 15.08 | <0.001 | 0.0011 | 0.0014 |
| Baseline mean weekly impact | 13.92 | 2.57 | 5.41 | <0.001 | 8.87 | 18.96 |
| Time (weeks) | 0.003 | 0.005 | 0.63 | 0.527 | -0.01 | 0.01 |
| Holiday/4-day week | -0.16 | 0.05 | -3.24 | 0.001 | -0.25 | -0.06 |
| Constant | 1.35 | 0.19 | 7.27 | <0.001 | 0.99 | 1.72 |
| Secondary analysis: Mixed model for log-transformed weekly revenue by site (N=54) | | | | | | |
|  | Coef. | Std. Err. | z | P-value | [95% Conf. | Interval] |
| Ecolabels present (intention-to-treat) | -0.04 | 0.01 | -2.78 | 0.005 | -0.07 | -0.01 |
| Total Quantity | 0.001 | 0.0000 | 16.54 | <0.001 | 0.0006 | 0.0007 |
| Time (weeks) | 0.01 | 0.002 | 5.19 | <0.001 | 0.01 | 0.01 |
| Holiday/4-day week | -0.22 | 0.02 | -12.80 | <0.001 | -0.26 | -0.19 |
| Constant | 7.67 | 0.08 | 94.22 | <0.001 | 7.51 | 7.83 |
| Secondary analysis: Mixed model for log-transformed weekly quantity of items sold by site (N=54) | | | | | | |
|  | Coef. | Std. Err. | z | P-value | [95% Conf. | Interval] |
| Ecolabels present (intention-to-treat) | 0.003 | 0.03 | 0.09 | 0.926 | -0.054 | 0.060 |
| Time (weeks) | 0.002 | 0.003 | 0.48 | 0.633 | -0.005 | 0.008 |
| Holiday/4-day week | -0.30 | 0.03 | -8.89 | <0.001 | -0.37 | -0.24 |
| Constant | 5.74 | 0.13 | 44.35 | <0.001 | 5.48 | 5.99 |

Supplementary Image 1. Example of a cafeteria display with the menu (with ecolabels), informational sheet, and visual display of the foods available.


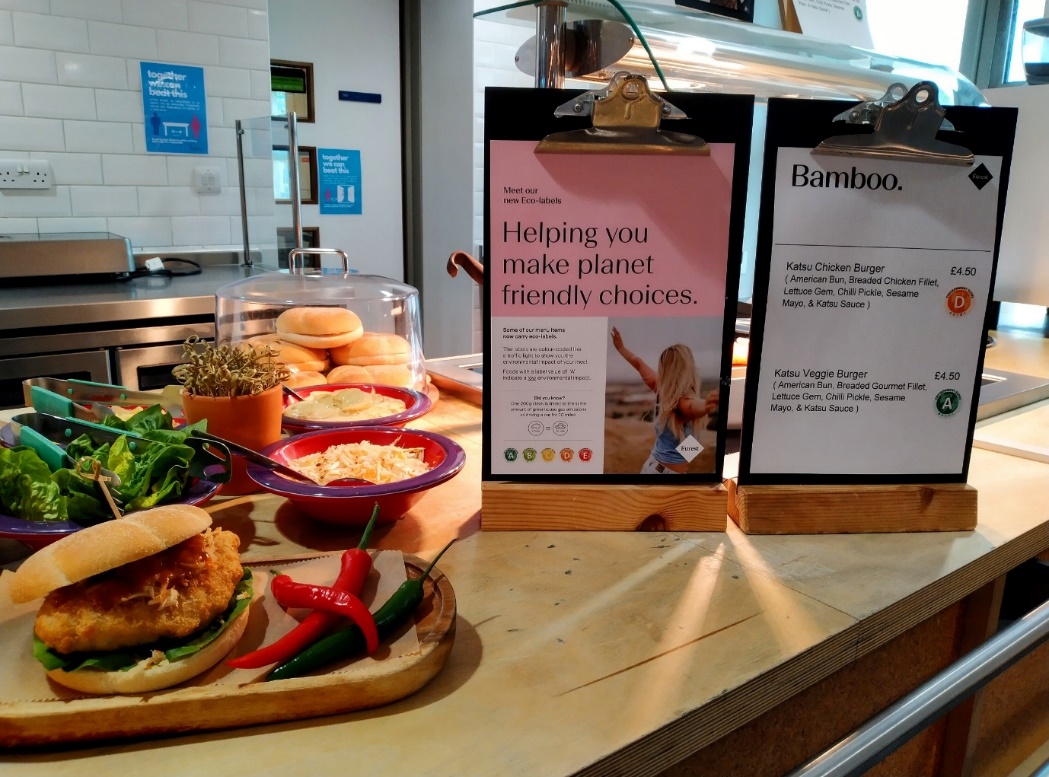

Supplement: Supplementary file 2 — Supplementary Material 2 [file 12889_2024_21272_MOESM2_ESM.docx]
